# Personalizing Personalized Medicine: The Confluence of Pharmacogenomics, a Person’s Medication Experience and Ethics

**DOI:** 10.3390/pharmacy11030101

**Published:** 2023-06-15

**Authors:** Timothy P. Stratton, Anthony W. Olson

**Affiliations:** 1 University of Minnesota College of Pharmacy, Department of Pharmacy Practice and Pharmaceutical Sciences, Duluth, MN 55812, USA; 2 Essentia Institute of Rural Health, Duluth, MN 55805, USA; anthony.olson@essentiahealth.org

**Keywords:** health care ethics, medication experience, person-centered care, pharmacogenomics, pharmaceutical care

## Abstract

Truly personalized precision medicine combines pharmacogenomics (PGx), a person’s lived medication experiences and ethics; person-centeredness lies at the confluence of these considerations. A person-centered perspective can help inform PGx-related treatment guidelines, shared decision-making for PGx-related therapeutics and PGx-related healthcare policy. This article examines the interplay between these components of person-centered PGx-related care. Ethics concepts addressed include privacy, confidentiality, autonomy, informed consent, fiduciary responsibility, respect, the burden of pharmacogenomics knowledge for both the patient and healthcare provider and the pharmacist’s ethical role in PGx-testing. Incorporating the patient’s lived medication experience and ethics principles into PGx-based discussions of treatment can optimize the ethical, person-centered application of PGx testing to patient care.

## 1. Introduction

The National Cancer Institute defines “precision” or “personalized” medicine as “a form of medicine that uses a person’s own genes or proteins to prevent, diagnose or treat disease” [1]. However, for precision drug treatment or pharmacotherapy to be truly “personalized”, at least two additional concepts need to be added to the calculus: treating the patient as a capable person [2], and accounting for a person’s lived experiences with medications [3].

Tomaselli and colleagues [2] argue that the concept of *patient*-centered care [4] is focused more on diagnosis and medical treatment, whereas *person*-centered care is based on relational ethics [5], seeing the person as an active collaborator in treatment decisions based on the person’s needs, family, history and capabilities. While relational ethics emerged from feminist ethics [6] (chap. 4–10), the pillars of relational ethics—mutual respect, engagement, embodied knowledge, environment and uncertainty [5]—encompass several bioethics concepts that will be discussed in this review. The connections between relational ethics and bioethics are shown in Table 1.

**Figure 1:**
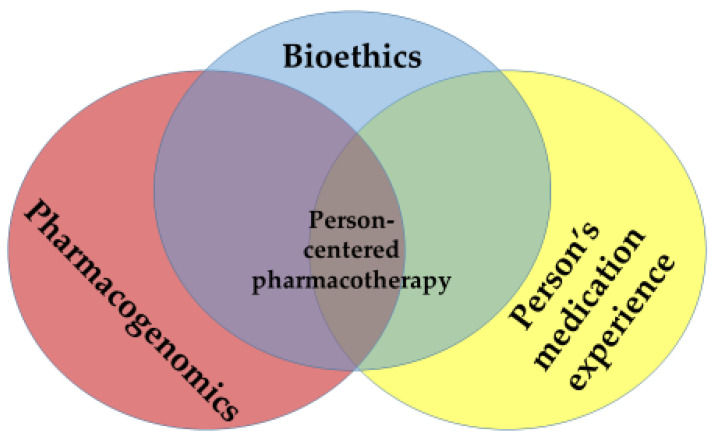
Person-Centered Personalized Pharmacotherapy: The Intersection of Pharmacogenomics, a Person’s Medication Experience and Bioethics.

Tomaselli’s conceptualization of person-centered care applies directly to pharmacotherapy decisions. Hillman et al. [7] note that a person’s attitudes and behaviors towards the use of medications are attenuated by how the person relates to health conditions that have afflicted themselves, family or influential others, and by experiences that they, their family or influential others have had with medications. Viewed as a Venn diagram (Figure 1), truly “person-centered” pharmacotherapy treatment decisions lie at the confluence of pharmacogenomics (PGx), a person’s medication experience and bioethics.

Significant overlaps occur between the respective dyads within the Venn diagram. The emerging sciences of genetic testing and PGx raise simultaneously important ethical questions for patients, healthcare and society [8,9]. Relational ethics considerations (mutual respect, engagement, embodied knowledge, environment and uncertainty) [5] can also influence—and be influenced by—a person’s medication experience [3]. Finally, a person’s genetic profile might heavily influence the experiences a person has with medications [10,11,12].

The purpose of this article is to examine the interplay between bioethics and the other components of the Venn diagram, and to demonstrate how these components—when considered in total—converge on a person-centered approach to making PGx-based pharmacotherapy treatment decisions. This intersection can be useful in informing PGx-related protocols for care, in guiding shared decision-making for PGx-related therapeutics, and in promulgating PGx-related healthcare policies that address individual as well as population-level considerations. This examination also aims to show that the core principles of relational ethics retain their broad applicability across situational uses of PGx (e.g., pre-emptive testing in healthy adults, finding the optimal medication for a new cancer diagnosis, choosing the most appropriate warfarin dose). The following discussion pertains to persons who are old enough to seek out their own health care without parental notification (N.B., the age at which this right can be legally exercised may vary from state to state) [13].

## 2. Bioethics Considerations Related to Pharmacogenomics and a Person’s Medication Experience

### 2.1. Privacy

As some similarities exist between PGx testing (i.e., testing how someone’s body processes medications) and traditional diagnostic genetic testing (i.e., testing whether someone has, or is at risk for developing, a genetic disease), several similar privacy concerns can also arise. A person choosing to undergo diagnostic genetic testing has the right to make informed, independent decisions about whether—and which—others may know details about the person’s genome (e.g., insurers, employers, educational institutions, spouses and other family members, researchers, and social agencies) [14] (p. 249).

Conversely, PGx testing differs from diagnostic genetic testing in that PGx testing is not intended to, and generally does not, reveal information about the risk of a person developing a particular disease [15]. For example, while PGx testing for pharmacokinetic and pharmacodynamic markers for certain psychiatric medications is currently available [16], at the time of this writing there is no single genetic marker or group of markers than can reliably diagnose a particular psychiatric illness [17]. As PGx testing is not used to diagnose, PGx testing raises fewer privacy concerns than diagnostic genetic testing might.

From a legal standpoint, the Genetic Information Nondiscrimination Act of 2008 (GINA) was promulgated to protect residents of the United States of America from discrimination based on their diagnostic genetic or PGx information when seeking either health insurance (Title I) or employment (Title II) [18]. In 2013, GINA amended the Health Information Portability and Accountability Act (HIPAA) of 1996 to clarify that genetic information is health information. The sharing and use of genetic information are therefore subject to the same HIPAA rules as any other protected health information [18]. Furthermore, employer-provided health insurance plans are prohibited from charging more to persons for which a pre-existing condition might be revealed through genetic testing. However, while GINA protects against genomic discrimination in obtaining health insurance and employment, these protections do not extend to a person’s ability to obtain life, disability or long-term care insurance [19].

While a person’s medication experience may not be explicitly categorized as protected health information, the privacy of one’s medication experience intersects with how the concept of privacy relates to a person’s PGx data. Hillman and colleagues point out that a person’s use of medications involves their perceived risks and concerns (i.e., vulnerabilities), which can extend beyond the biomedical realm and into the social realm [7]. For example, employing PGx to tailor treatments for substance use disorder (the biomedical realm) may leave a person more vulnerable to additional stigmatization (the social realm) because of genetic links between addiction risk and certain psychiatric conditions [20]. This makes a person’s right to share or not share information about their experiences with drugs that have addiction potential and/or experiences with medication-assisted therapy to treat addiction very impactful on their day-to-day life.

### 2.2. Confidentiality

Related to maintaining the privacy of a patient’s genetic or PGx information is the ethics concept of confidentiality [14] (p. 3). Maintaining the confidentiality of a patient’s PGx information arises from the ethics concepts of autonomy (discussed below), fidelity (the healthcare provider’s promise not to share a patient’s PGx information without the patient’s permission, except in extenuating circumstances, as discussed below) and right to privacy, as discussed previously.

Maintaining the confidentiality of a patient’s data, however, is not absolute. The healthcare provider may ethically breach confidentiality where such breach is necessary to avert serious harm to others [14] (p. 16). However, what if this “other” is a first-degree relation who may share much of the patient’s genome? Disclosing a patient’s genetic test results to a family member without the patient’s consent remains problematic, as noted by Callier and Simpson in the *AMA Journal of Ethics* [21]:

- The threat to family members is rarely imminent and the level of foreseeable harm is often difficult to predict;
- Personalized genomic medicine complicates what it means to act in the best interest of the patient; variations in family dynamics can quickly and dramatically transform the fulfillment of professional duty to the patient to a questionable act for a family member;
- Genetic test results often provide only probabilistic information rather than a clear diagnosis or definite prediction of disease;
- It is unclear whether relatives should be warned of hereditary conditions when there are no means of prevention, treatment, or cure;
- There is little support for warning underage family members of adult-onset conditions;
- Patients’ relatives also have a “right *not* to know” about their genetic makeup, so informing them might interfere with their autonomy, in addition to breaching the patient’s confidentiality.

As PGx is not the same as diagnostic genetic testing, then based on the concerns outlined by Callier and Simpson, notifying a family member about the patient’s PGx results would be ethically *even less* justified than notifying the family member about the patient’s diagnostic genetic testing results.

### 2.3. Autonomy

Closely related to decisional privacy is the ethical precept of autonomy [14] (p. 248), which recognizes that rational (competent) persons must be given the choice to make their own decisions. To make an informed, autonomous decision, a person must also have information about the potential consequences of their actions, a principle underlying the concept of “informed consent” (discussed later in this article). As noted in the discussion about privacy, a person has the right—the autonomy—to choose who may have access to information related to that person’s genetic tests.

A person’s medication experience also informs their autonomous decisions, related to the person’s ambivalent attitude towards using a particular medication at a certain time to treat a certain condition, or not [7]. The person may realize that there could be potential benefits from using a medication, such as relief from symptoms, improved functionality, or maintaining or enhancing well-being. These potential benefits, however, might be offset or even outweighed by potentially deleterious aspects of using the medication. Undesirable consequences may be directly related to the medication itself, such as adverse effects from the medication. Alternatively, undesirable consequences may be indirect, such as financial out-of-pocket “opportunity costs” [22] of the medication—forgoing the benefit(s) from other desired purchases or life necessities for the sake of purchasing the medication. Another indirect undesirable consequence of using a particular medication may be lifestyle changes necessitated by the medication, such as permanent changes to one’s diet or discontinuing one’s favorite activities.

### 2.4. Informed Consent

Informed consent is a hallmark of person-centered care [23]. As noted above, for a competent individual to make an autonomous decision about undergoing PGx testing and/or proceeding with treatment, they need to be fully informed about the potential consequences of PGx testing versus not testing, as well as the potential consequences of different treatment options versus no treatment. Informed consent is a process of the person receiving education and having the opportunity to have questions answered, not just signing a consent form [14] (p. 220) [24].

Informed consent also protects the patient from being subjected to paternalistic provider decisions (or parentalistic, as female providers can be just as “paternalistic” as male providers). The informed consent process is a tacit recognition that the patient is participating as an active partner with the provider in a shared decision about whether the patient will undergo testing or receive treatment, rather than the provider compelling the patient to participate in these activities [25].

Applying the concept of informed consent to PGx testing, a person deserves to have information provided about the following (modified from the University of Oregon) [26]:

- An explanation of the purposes of the testing;
- The testing procedure to be followed;
- A description of any reasonably foreseeable risks from testing;
- A description of any benefits the person may reasonably expect from the testing;
- Disclosure of appropriate alternative procedures, testing or treatments that might be advantageous to the person;
- A statement describing the extent to which confidentiality identifying the person will be maintained;
- A statement that the person’s PGx information, even if identifiers are removed, will not be used or distributed for future research studies without the person’s permission;
- A statement that participation in PGx testing is voluntary, and that the person may decline or change their mind without penalty or loss of benefits to which the person is otherwise entitled;
- Any additional costs that the person may incur from PGx testing.

Regarding a person’s medication experience, informed consent, as noted in the Autonomy section above, is imperative to enable a person to make an informed decision about whether to use a specific medication or not. A healthcare professional withholding information to steer a patient towards a course of action preferred by the professional is parentalistic, unethical behavior [24]. Consider a person who has high blood cholesterol levels that warrant medication therapy [27]. The patient is reluctant to start statin therapy because a friend who had used “some kind of statin” to treat their high cholesterol had developed muscle pain and weakness. The healthcare provider notes that the chance of this side effect occurring is very low [27,28], but to provide the patient peace of mind, offers PGx testing for the *SLCO1B1* gene [29]. However, non-statin treatment approaches can also be used to reduce blood cholesterol levels [30], and if these options are not also offered to the patient, the healthcare provider may be denying the person the opportunity to make a truly informed decision.

### 2.5. Fiduciary Responsibility: Assessing Potential Benefits and Harms

Healthcare providers have a fiduciary responsibility to their patients. This responsibility arises from the power imbalance that exists because the healthcare professional has expertise in areas of importance to the patient, about which the patient knows little or nothing. In such instances, the patient must rely on the healthcare professional’s advice and actions [7] (p. 7) [31]. Neuroscientist and ethicist Làzaro-Muñoz notes that among a clinician’s fiduciary duties related to diagnostic genomic testing is the duty to respect a patient’s self-determination; however, he further notes that protecting a patient’s autonomy rights can be at odds with the capabilities promised by genomics [19].

Although Làzaro-Muñoz’s discussion specifically addresses diagnostic genomic testing rather than PGx testing, a question relevant to PGx testing arises: who should assess the benefit/harm ratio of PGx testing? Should that risk/benefit evaluation be undertaken by the clinician, who generally knows a great deal more about interpreting PGx test results than the patient? Or should such an assessment be made by the patient, who ultimately would need to decide whether they would even want a PGx test in the first place, let alone decide how to act on the results from such a test?

This difficult act of balancing a healthcare provider’s knowledge and maintaining the patient’s right to make their own decisions can be further exacerbated by the patient’s medication experience. Revisiting the statin-related patient scenario presented in the previous section, the medication experience of the patient’s friend may be so compelling in the patient’s mind that the patient continues to refuse to have anything to do with statin therapy, despite the overwhelming evidence regarding the safety of statin therapy [28] and the assurance the person might gain by undergoing PGx testing for the *SLCO1B1* gene [29].

### 2.6. Respect for Persons

One of the foundational concepts underlying patient-centeredness is having respect for patients as persons [32]. This fundamental concept is based on the ethics precept of German philosopher Immanuel Kant that human beings be treated in a fashion that respects the special moral status persons have, including the person’s right to make autonomous decisions [33,34]. When a person becomes a patient, they already lose some of their “personhood”, tending to be subcategorized by age, sex, clinical condition, race and/or comorbidity. PGx adds an additional level of categorization, based on a patient’s genetic disposition to potentially impact the metabolism of, and response to, specific drugs.

There are certainly advantages to PGx testing, such as providing more information to improve shared treatment decision-making, and potentially improve treatment and health outcomes. From a clinical research perspective, pharmacogenomically defining clinical trial participants can enable smaller trials to be conducted, as only potentially positive responders would participate. In addition to potentially reducing the rate of adverse effects among study participants, selecting study participants based on pharmacogenomic profiling could also ultimately reduce the number of participants who drop out of a trial due to intolerance to the study drug.

PGx categorization, however, might also lead to a patient being denied a specific treatment based on their genetic characterization, such as a person who has breast cancer being denied tamoxifen therapy, as PGx testing for the CYP2D6 allele suggests that the person is a genetically poor metabolizer of tamoxifen [35]. PGx categorization may also lead to a person being labeled for life as “difficult to treat” [36]. However, by adopting a patient-as-person perspective, i.e., conceiving of the person “as an experiencing individual rather than the object of some disease” [37], the healthcare professional can “inoculate” themselves against perceiving the person as “difficult to treat.”

A person’s PGx profile based on a person’s race can be either helpful or detrimental. While race is a social construct [38], there are indeed instances where differences in drug response are genetically associated with a person’s race:

- An increased rate of irinotecan-related adverse effects has been found to occur in persons of sub-Saharan African descent [12].
- An increase in carbamazepine-related adverse reactions has been found to occur in persons of Southeast Asian descent [10].
- Therapeutically significant differences in responses to medications used to treat cardiovascular disease have been found between patients of different racial backgrounds [11].

Race-based “differences” in drug therapy response, however, have also been misused. In 2005, NitroMed received FDA approval for BiDil^®^ (hydralazine HCl and isosorbide dinitrate) to be used in African American patients to treat congestive heart failure. The US Food and Drug Administration (FDA) initially rejected the new drug application for BiDil^®^ as the product failed to show a statistically significant benefit over placebo in a multiracial population [39]. However, upon the recommendation of an FDA advisory committee, the company subsequently undertook a new clinical trial among 1050 men and women who self-identified as African American and who had a diagnosis of congestive heart failure. Patients in this trial who received BiDil^®^ experienced 39% fewer first hospitalizations and 43% fewer deaths than patients enrolled in the placebo group. Based on the results of this and other clinical trials that demonstrated the benefit of BiDil^®^ in Black patients, BiDil^®^ was approved by the FDA for use in Black patients who had cardiac disease [39]. Patients enrolled in these trials, however, had self-identified as a particular racial group rather than any specific genetic marker being used, and no specific racial gene is necessarily linked to ancestry or skin color [39].

## 3. Burden of Knowledge, Uncertainty and the Patient Medication Experience

As alluded to in the privacy section, in addition to providing information about a patient’s genetic likelihood of positively responding to a particular drug or suffering a genetically linked side effect, PGx testing may also unintentionally provide information pertaining to the patient’s genetic predisposition for developing specific diseases or conditions, provide prognostic information or provide information applicable to other classes of drugs not currently prescribed for the patient. More than 50% of 42 PGx tests associated with drug response were also reported to be associated with diseases [40]. For example, the APOE4 allele associated with decreased warfarin dose requirements can also provide risk information about Alzheimer’s disease [41]. Haga and colleagues [41] have termed this phenomenon the “informational side effect” of PGx testing.

However, what if a patient does not want to know this additional genetic information about themselves? This has been termed “the burden of knowledge” [42]. While a patient has a right to know their pharmacogenomic information, in order to make informed decisions about their health, they also have the right to NOT know that information. A patient may not want to know if they have a chance of developing a debilitating disease for which no treatment is available. In addition, having the genetic variation does not necessarily mean that the person will go on to develop the disease or condition; the predictive value of the test may be suspect, and preventative measures may exist that every person would want to implement whether they had the genetic variation or not [8].

This burden of knowledge could also impact the healthcare provider. Consider the patient whose pharmacogenomic profile also suggests that the patient is at risk for developing a serious condition or disease. The patient may not wish to notify first-degree family members who carry that same genetic risk, nor might the patient consent to allow the family members to be notified by the healthcare provider. The ethical conflict in this scenario arises from a conflict between the patient’s personal values and the healthcare provider’s professional values. Whereas the patient may wish to keep information about their health confidential and refuse to share this pharmacogenomic information with family members, the healthcare provider may believe that the potential harm to family members from not disclosing is greater than the harm to family dynamics from violating the patient’s privacy by disclosing. Indeed, in such instances the American Society of Human Genetics has opined that the healthcare provider should breach patient confidentiality [43,44]. Other important ethical considerations related to this scenario include the patient identifying at-risk family members without first receiving permission from the family members to be identified or contacted.

The burden of knowledge can also simultaneously influence several different attributes of a person’s attitudes towards a medication by increasing the person’s uncertainty about that medication [7]. Consider once again the person in the statin scenario described earlier. The influence of what the person knows, or perceives that they know, about how statin therapy might impact their life is summarized in Table 2. In some cases, this knowledge can influence multiple attributes of a person’s medication experience in contradictory ways.

## 4. Person-Centered Shared Decision-Making Regarding Whether to Undergo PGx Testing

For a person considering whether or not to undergo PGx testing, they must know specific information about genetics, the test, the disease or condition and the potential treatment. How prevalent is it, and what is the penetration of the gene variation? That is, epidemiologically, how likely is the patient to have the gene variation being tested for? How likely is the PGx test to produce a false negative (sensitivity) or false positive (specificity) result? How much will the test cost the patient out-of-pocket? Will the results from the PGx test be obtained in a timely fashion? If undertreated or left untreated, how likely is the condition or disease to result in significant morbidity or mortality? If the person is found to have the genetic variation of interest, what is the prevalence of the associated pharmacokinetic variation in response or adverse effects [45,46,47]? In working with the patient to find answers to these questions, the healthcare provider is participating in person-centered care at the highest level, functioning as the person’s fiduciary while respecting the person’s autonomy to make the final decision to test or not to test.

## 5. The Pharmacist’s Role in PGx Testing and in the Patient’s Medication Experience

Pharmacists bring a great deal of specific expertise to the discussion about PGx testing [48]. At the time of writing, relative to most other healthcare professionals, pharmacists possess an increased understanding of genetic factors that impact drug safety and efficacy [49,50,51,52]. They have enhanced education about the genetic etiology of drug responses, PGx instruction being required in all US pharmacy programs since 2016 [53]. Informed by the principles of relational ethics highlighted previously, pharmacists are in an excellent position to assist prescribers in considering PGx test results [48,49,50] in instances such as selecting oncology treatments [54] or adjusting warfarin doses [55].

Pharmacists are also in an excellent position to educate patients regarding PGx testing, although patients may not always be aware of the pharmacist’s expertise in this area [54]. To maximize their effectiveness in the role of “PGx counselor”, pharmacists need to discuss PGx testing in the context of the patient’s medication experience [7] (pp. 10–11). In a focus group interview with pharmacists providing medication therapy management (MTM) services [54], participants noted that most patients had preconceived ideas about medications, shaped by patients’ past experiences or the experiences of others. Therefore, pharmacists needed to be attentive to these subjective experiences if they were to help patients maximize the benefit they would receive from their medications. This approach takes on added importance when discussing new technologies such as PGx with patients, as patients may have incomplete or inaccurate information, or may have misinterpreted information they obtained from the lay press, the Internet, or from social media [56].

There are also potential legal considerations for the pharmacist regarding the intersection of PGx, the patient’s medication experience and bioethics. Under the provisions of the Omnibus Budget Reconciliation Act of 1990 (OBRA 90), if the pharmacist is aware of or learns that a person with a particular genetic variant may be at risk of an adverse event but does not inform the patient of this possibility, that pharmacist could be found in breach of their duty to the patient [9].

## 6. Summary

When providing person-centered care, it is impossible to separate clinical considerations from ethical considerations. This reality becomes more complicated when discussing the use of new technologies such as PGx testing, for which ethical considerations may not have been adequately addressed prior to fully deploying the technology. Incorporating PGx knowledge, the patient’s lived medication experience and relational and bioethics principles into discussions of testing and treatment as part of a shared decision-making process involving prescribers, pharmacists and patients can optimize a person-centered approach to the use of PGx testing.

## Figures and Tables

**Table 1:** Connections between Relational Ethics concepts and Bioethics concepts.

Relational Ethics Concepts [5]	Relevant Bioethics Concepts
Mutual respect	Respect for persons Beneficence Non-maleficence Privacy Confidentiality Autonomy
Engagement (Establishing a patient–provider relationship)	Beneficence Privacy Confidentiality Informed consent Fiduciary responsibility
Embodied knowledge (Patient’s lived experiences)	Non-maleficence Autonomy Informed consent
Environment (Patient’s needs, preferences, values, family, community, history)	Non-maleficence Justice
Uncertainty (Decisions based on different value-based demands)	Autonomy Burden of knowledge

**Table 2:** Influence of the burden of knowledge on the medication experience of a person who has newly diagnosed elevated cholesterol.

Medication Experience Attribute [7]	Influence of Knowledge
Ambivalence	I have a friend who suffered side effects from a similar medication Are the cost and inconvenience of taking a medication for years or decades worth preventing a heart attack? I have friends who suffered a heart attack—not good!
Vulnerability	Could I suffer similar medication side effects? I need to lower my LDL cholesterol to avoid a cholesterol-related cardiac event I would rather not take a medication for the rest of my life
Pragmatism	I would rather feel well than have a medication side effect I would rather feel well than suffer a heart attack I would rather not take medication daily for the rest of my life
Context and nuance	I feel good and am not feeling any effects from having high cholesterol I have friends who suffered a heart attack—not good! I do not like having to take medications at all, let alone every day
Active ongoing process	I would rather not need to take a medication for the rest of my life

## Data Availability

No new data were created or analyzed in this study. Data sharing is not applicable to this article.
